# Single-cell immune landscape of the central nervous system of mice infected with rabies virus

**DOI:** 10.3389/fimmu.2026.1850356

**Published:** 2026-07-02

**Authors:** Xinyue Wang, Xinjie Zhang, Wenwen He, Xianzhu Xia, Pingsen Zhao

**Affiliations:** 1 College of Wildlife and Protected Area, Northeast Forestry University, Harbin, China; 2 Department of Laboratory Medicine, Yuebei People’s Hospital Affiliated to Shantou University Medical College, Shaoguan, China; 3 Laboratory for Diagnosis of Clinical Microbiology and Infection, Yuebei People’s Hospital Affiliated to Shantou University Medical College, Shaoguan, China; 4 Research Center for Interdisciplinary & High-quality Innovative Development in Laboratory Medicine, Shaoguan, China; 5 Shaoguan Municipal Quality Control Center for Laboratory Medicine, Yuebei People’s Hospital Affiliated to Shantou University Medical College, Shaoguan, China; 6 Shaoguan Municipal Quality Control Center for Surveillance of Bacterial Resistance, Yuebei People’s Hospital Affiliated to Shantou University Medical College, Shaoguan, China; 7 Shaoguan Engineering Research Center for Research and Development of Molecular and Cellular Technology in Rapid Diagnosis of Infectious Diseases and Cancer, Yuebei People’s Hospital Affiliated to Shantou University Medical College, Shaoguan, China; 8 College of Veterinary Medicine, South China Agricultural University, Guangzhou, China; 9 Changchun Veterinary Research Institute, Chinese Academy of Agricultural Sciences, Changchun, China

**Keywords:** central nervous system, immune response, pathogenicity differences, RABV, scRNA-seq

## Abstract

**Background:**

The fatality rate of virulent rabies virus (RABV) following central nervous system (CNS) invasion is nearly 100%. Infection with the virulent CVS-11 strain is associated with severe neurological symptoms and lethal outcomes, while the attenuated SRV9 strain can be cleared by the host. Although previous studies have investigated intracranial cytological and immunological changes, a high-resolution single-cell understanding is still lacking. Such resolution is essential for analyzing immune cell heterogeneity and intercellular communication networks.

**Objectives:**

This study aimed to depict the immune response after CVS-11 and SRV9 infections at single-cell resolution, addressing the immune mechanisms influencing RABV infection outcomes.

**Methods:**

We performed single-cell RNA sequencing (scRNA-seq) on brains from CVS-11 infected, SRV9 infected, and mock infected mice. By analyzing over 100,000 cells, we constructed a comprehensive atlas of CNS immune responses.

**Result:**

Compared with SRV9 infection, CVS-11 infection was associated with transcriptional signatures indicative of: a trend of microglial shifting toward a phagocytic signature enriched phagocytic phenotype, elevated expression of genes related to excessive neutrophilic inflammation, down regulation of NK cell functional genes (suggesting potential dysfunction), and increased expression of T cell exhaustion-related genes. In contrast, SRV9 infection correlated with microglial features indicative of an immunoregulatory phenotype, more precise NK cell antiviral function, more complete T cell activation and memory formation, and more coordinated immune interactions.

**Conclusion:**

The scRNA-seq data from this study suggest that virulent and attenuated RABV strains may induce distinct patterns of immune responses in the central nervous system: the former is accompanied by features of dysfunctional cellular responses, whereas the latter presents protective immune features associated with viral clearance. Notably, a set of signature genes (*Fkbp5, Apod, Klf2, Socs3*) and pathways was identified associated with lethal RABV infection. These findings provide new insights for rabies vaccine design and immunotherapy.

## Introduction

RABV (rabies virus) is a typical neurotropic virus. Once clinical symptoms appear, the fatality rate approaches 100% (1). Despite the availability of effective pre-exposure and post-exposure prophylaxis, rabies remains a major public health threat in many developing countries, causing tens of thousands of deaths each year (2, 3). The pathogenesis of rabies is closely related to the virus’ ability to invade the central nervous system and disrupt the host’s immune response (4, 5). CVS-11 is a laboratory-fixed virulent RABV strain with high pathogenicity, the virus spreads rapidly, leading to severe neurological symptoms and a 100% mortality rate (6–8). In contrast, the laboratory-fixed SRV9 strain can be effectively cleared from the central nervous system (CNS), resulting in improved survival rates in mice, and is therefore commonly referred to as an attenuated strain. The distinct phenotypic outcomes following infection with these two strains provide a comparative model for investigating the determinants of rabies pathogenicity and protective immunity (9).

Previous studies have made significant progress in characterizing the cytological and immunological changes in the brain following RABV infection (10). It has been reported that RABV can infect not only neurons but also astrocytes and even infiltrating myeloid cells, suggesting that remodeling of the central nervous system immune microenvironment may be linked to viral pathogenicity (4, 11, 12). Furthermore, existing evidence indicates that T cell responses play a critical role in viral clearance, whereas RABV employs multiple immune evasion strategies, including the induction of immune cell apoptosis and the expression of immunosuppressive molecules (13, 14). While our previous work delineated global transcriptomic and alteration of miRNA profiles in RABV infected mouse brains at the bulk-tissue level, the cell-type-specific immune dynamics driving divergent pathogenic outcomes remain unresolved (15, 16). However, most previous studies used bulk sequencing, flow cytometry, or targeted assays. These methods lack sufficient resolution. They cannot fully define immune subset heterogeneity, dynamic transcriptional states, or intercellular networks in the CNS (17).

Specifically, the coordinated response patterns of distinct immune lineages, including microglia, myeloid cells, NK cells, and T cells, during RABV infection remain incompletely understood (18). The transcriptional programs that drive microglia toward protective or pathological activation states have yet to be clarified (19). Whether and how highly virulent RABV infection actively induces T cell exhaustion, and the molecular features of such a process, remain to be determined. Moreover, differences in intercellular communication networks between lethal and non-lethal infections have not been systematically characterized. In recent years, advances in single-cell RNA sequencing have enabled the analysis of cellular heterogeneity and transcriptional states in complex tissues, offering opportunities to systematically map the central immune landscape during RABV infection and to identify cellular and molecular features that distinguish virulent from protective immune responses (11, 20, 21).

In this study, we performed single-cell RNA sequencing (scRNA-seq) on whole brain immune cells from mice infected with CVS-11, SRV9, or control DMEM (Dulbecco’s Modified Eagle Medium).Three experimental groups were established in this study, including CVS-11 intracerebral infection (CVic) group, SRV9 intracerebral infection (SRic) group, and DMEM mock intracerebral injection (DMic) control group. By integrating the transcriptional profiles of over 100,000 cells, we constructed a comprehensive atlas of central immune responses induced by RABV strains with differing pathogenicity. This study aimed to observe global changes in immune cell composition induced by virulent and attenuated infections, to characterize the activation status and transcriptional programs of key immune lineages, including microglia, myeloid cells, NK cells, and T cells, and to explore the molecular pathways and intercellular communication networks that may underlie the divergent outcomes following infection with virulent and attenuated strains. These analyses were intended to provide a high resolution reference for understanding the neuroimmune mechanisms of RABV pathogenesis and to offer a foundation for the rational design of vaccines and immunotherapeutic strategies.

## Materials and methods

### Mice and infection

We have clearly clarified that 6–8 week old female C57BL/6 mice were used in this study. The detailed viral inoculation procedure: mice were stereotactically inoculated intracranially with 2 × 10^6^ TCID_50_ (50% tissue culture infectivedose) of RABV (2 μL) at the right lateral ventricle (X = 1.00 mm, Y = 0.80 mm, Z = 1.5 mm). Equal volumes of Dulbecco’s Modified Eagle Medium were injected into mice in the mock group using the same procedure. The CVS-11 and SRV9 rabies virus strains were kindly provided by the Changchun Veterinary Research Institute, Chinese Academy of Agricultural Sciences, and both strains have undergone consistent standard laboratory passages. For survival analysis, 10 mice per group were monitored for 10 days post-infection. For body weight and clinical score assessment, 4 mice per group were used.Clinical signs were scored daily using a 0−5 scale as established in our laboratory following standard protocols: 0, no clinical signs; 1, movement disorders; 2, ruffled fur and hunched posture; 3, tremors and shivering; 4, complete loss of mobility or total paralysis; 5, death. Humane endpoint criteria were predefined as mice reaching a clinical score of 4 (complete loss of mobility or total paralysis) or exhibiting body weight loss exceeding 20%; such mice were humanely euthanized immediately by cervical dislocation under deep isoflurane anesthesia (5% induction).

### Anesthesia and euthanasia

For all surgical procedures and tissue collection, mice were anesthetized by inhalation of isoflurane (RWD Life Science, China) delivered in 100% oxygen at a flow rate of 1 L/min. Anesthesia was induced with 3% isoflurane and maintained with 1.5% isoflurane via a nose cone. Depth of anesthesia was verified by the absence of pedal withdrawal reflex. After transcardial perfusion or brain dissection, mice were euthanized by cervical dislocation under deep isoflurane anesthesia (5% induction). For mice meeting humane endpoint criteria (clinical score of 4 or body weight loss >20%), euthanasia was performed immediately by cervical dislocation without prior perfusion.

### FAVN method for detecting rabies neutralizing antibodies in serum

Serial blood samples were collected from the same individual mice via the retro-orbital vein on days 1, 3, 5, 8, and 10 post-infection, and serum was isolated from each sample (n=3). First, the test serum and WHO standard positive/negative control sera were inactivated at 56 °C for 30 min, then serially diluted starting from 1:3 with DMEM medium (the test serum was diluted to 1:6561). Meanwhile, log-phase BHK-21 cells were digested, adjusted to a concentration of 2×10^5^ cells/mL, seeded into 96-well plates, and cultured at 37 °C for 18–24 hours until a 100% confluent monolayer was formed. After the CVS-11 virus is titrated for TCID_50_, it is diluted to 100 TCID_50_ per 50 μL volume. Subsequently, 50 μL of the diluted serum and 50 μL of the virus solution were added to each well of the cell plate (virus control wells and cell control wells were set up) and incubated at 37 °C for 2 h to complete the neutralization reaction. Then, 100 μL of DMEM medium was added for further culture at 37 °C for 48–72 hours. Once the virus control wells showed full-well fluorescence, the cells were fixed with 4% paraformaldehyde, permeabilized with 0.1% Triton X-100, incubated with FITC-labeled anti-rabies nucleoprotein antibody(FUJIREBIO, Japan), washed. Finally, observation was performed using a fluorescence microscope; the highest dilution of serum that resulted in no fluorescent signal (complete inhibition of virus replication) was taken as the titer endpoint, and the neutralizing titer of the test serum (unit: IU/mL) was calculated with reference to the titer of the standard serum.

### Pathological analysis of mouse brain tissue

Brain tissues for histopathological analysis were uniformly collected at 5 days post-infection. We have explicitly defined the analyzed brain region as the dorsal one-third of the cerebrum in horizontal sections, which mainly contains the superficial cerebral cortex, cingulate cortex, and dorsal periventricular tissues. Mice were anesthetized with isoflurane (3% for induction, 1.5% for maintenance) in 100% oxygen at a flow rate of 1 L/min, then transcardially perfused with PBS. After perfusion, mice were euthanized by cervical dislocation under deep anesthesia. After dehydration and paraffin embedding of the brain tissue, 5 μm thick sections were prepared. The sections were stained with hematoxylin and eosin (H&E) for histopathological examination. A digital slide scanning system was used to acquire images and conduct pathological diagnostic observations. Green arrows indicate perivascular cuffing (multi−layered perivascular infiltration of inflammatory cells); yellow arrows indicate gliosis (increased local cell density with proliferating microglia/astrocytes); red arrows indicate neuronal necrosis (cell body shrinkage, pyknosis, and eosinophilic cytoplasm).

### Cell sorting and scRNA-seq

Adult C57BL/6 mice from each group were anesthetized with isoflurane (induction 3%, maintenance 1.5%) in 100% oxygen (1 L/min) and transcardially perfused with PBS. Following perfusion, mice were euthanized by cervical dislocation under deep anesthesia. Their brains were subsequently collected. Single-cell suspensions were obtained using the Adult Brain Dissociation Kit (Miltenyi Biotec, Cat. No. 130-107-677). Samples with cell viability ≥ 90% were used for 10× Genomics single-cell sorting and single-cell RNA sequencing, which was technically performed by Capitalbio Corporation (Beijing, China).

### Processing of single-cell RNA-seq data and quality control

Cell Ranger (version 6.1.2) was applied to filter low-quality reads, align reads to mouse reference genome (GRCm38), assign cell barcodes, and generate unique molecular identifier (UMI) matrices. The output gene expression matrices were analyzed by R software (version 4.3.1) with the Seurat package (version 4.4.0). Doublets predicted by Scrublet (version 0.2.1) were removed from each sample. And then all samples were merged into one Seurat object using the merge function in Seurat. Cells with fewer than 200 genes detected or fewer than 500 UMI counts detected or >10% mitochondrial UMI counts were filtered out.

### Dimensionality reduction, unsupervised clustering and cell-type annotation

The top 2,000 highly variable genes were detected using Seurat’s FindVariableFeatures function and retained for further dimensionality reduction.To reduce dimensionality of each cell, the RunPCA function was conducted with default parameters on linear-transformation scaled data generated by the ScaleData function. Next, the ElbowPlot function was used to identify proper dimensions of each dataset. After projection of all cells into two-dimensional space by RunUMAP function, we initially built a graph of cells by using the K-Nearest Neighbors (KNN) algorithm. Classification of each cell type was inferred from the cluster-specific genes. Doublets were identified by searching for cells with substantial and coherent expression profiles from two or more cell types. The main cell types were annotated based on the expression pattern of differentially expressed genes (DEGs) and the well-known cellular markers from the literature.

To identify subpopulations within these major cell types, we performed a second-round of unsupervised clustering. The second round clustering procedure was similar to the first-round clustering, which started from expression matrix of the subset of the major cell types, and then identified HVGs, calculated PCA (principal components analysis) matrix, corrected batch effects by Harmony, detected cell clusters by Louvain algorithm and performed dimensionality reduction for visualization. The number of principal components in each major subtype was independently determined by the Elbowplot function in Seurat. Cluster specific genes were detected using the FindAllMarkers function with default parameters.

### Gene set enrichment analysis

Differentially expressed genes (DEGs) between the CVic and DMic groups, as well as between the SRic and DMic groups, within the same cell subgroups were identified using the FindMarkers function provided by Seurat. For each comparison, genes with |log2FC| > 0.25 and adjusted *P value* < 0.05 were considered as DEGs. Subsequently, common up-regulated genes (upregulated in both CVic vs. DMic and SRic vs. DMic comparisons) and common down-regulated genes (downregulated in both comparisons) were extracted for functional enrichment analysis. Gene Ontology (GO) and Kyoto Encyclopedia of Genes and Genomes (KEGG) pathway enrichment analyses were performed using the R package cluster Profiler (version 4.2.2). The Benjamini-Hochberg (BH) method was applied for multiple test correction. GO terms and KEGG pathways with an adjusted *P value* < 0.05 were considered significantly enriched.

### Relative enrichment of cell clusters

We calculated the *R_o/e_* (the ratio of observed over expected cell numbers) for each cell cluster to quantify its relative enrichment or depletion across the three experimental groups (DMic, SRic, CVic). The expected cell numbers for each combination of cell cluster and experimental group were obtained from the chi-squared test.Our *R_o/e_* based analytical approach followed the framework established in our previous large-scale single-cell study of infectious diseases (22).

### Single-cell trajectory analysis

Monocle (Version 2.30.1) aims to resolve cellular transitions during differentiation through pseudotemporal profiling of scRNA-seq data. After inputting the count matrix into the “newCellDataSet” function with its clustering information, it was computed into a lower dimensional space based on the discriminative dimensionality reduction with trees (DDRTree) method using 2000 high variation genes by FindVariableFeatures function.

### Flow cytometric analysis

For flow cytometric analysis of brain immune cells, mice were anesthetized with isoflurane (3% for induction, 1.5% for maintenance) in 100% oxygen at a flow rate of 1 L/min at 5 days post-infection, and then transcardially perfused with ice-cold PBS. After perfusion, mice were euthanized by cervical dislocation under deep isoflurane anesthesia (5% induction). Whole brains were dissected and processed into single-cell suspensions using an Adult Brain Dissociation Kit (Miltenyi Biotec, Cat. No. 130-107-677). Non-specific Fc receptor binding was blocked with TruStain FcX™ PLUS (anti-mouse CD16/32) Antibody (Biolegend, Cat. No. 101320) prior to immunofluorescence staining. Cells were stained with fluorochrome-conjugated antibodies against APC/Cyanine7 anti-mouse CD45 Antibody (Biolegend, Cat. No. 157204), FITC anti-mouse/human CD11b (Biolegend, Cat. No. 101206), PerCP/Cyanine5.5 anti-mouse Ly-6C Antibody (Biolegend, Cat. No. 128012), BV605™ anti-mouse CD11c (Biolegend, Cat. No. 117333), PE Rat Anti-Mouse I-A/I-E (BD, Cat. No. 557000), APC Mouse Anti-Mouse NK-1.1(PK136) (BD, Cat. No. 550627), PE/Cyanine7 anti-mouse CD19 (Biolegend, Cat. No. 152418), and FITC anti-mouse CD3 (Biolegend, Cat. No. 100204). Dead cells were excluded using Zombie Aqua Fixable Viability Kit (Biolegend, Cat. No. 423101). Flow cytometry data were acquired on a flow cytometer and analyzed using FlowJo software (version 10.9.0). Gating was performed sequentially as follows: single cells, live cells and CD45+ leukocytes, followed by identification of monocyte/macrophages, dendritic cells, NK cells, and T cells.

### Statistics

All statistical analyses were performed using R software (version 4.3.1) unless otherwise specified. For single-cell RNA-seq data, differentially expressed genes (DEGs) between experimental groups (e.g., CVic vs. DMic, SRic vs. DMic, and CVic vs. SRic) were identified using the FindMarkers function in the Seurat package (version 4.4.0) with the Wilcoxon rank-sum test. To correct for multiple hypothesis testing, the Benjamini-Hochberg (BH) method was applied, and genes with an adjusted *P value* (adj.*P.Val*) < 0.05 and |log_2_ fold change| > 0.25 were considered statistically significant DEGs. For Gene Ontology (GO) and KEGG pathway enrichment analyses, the hypergeometric test was used, and the BH procedure was again applied to control the false discovery rate (FDR); terms with an adjusted *P value* < 0.05 were considered significantly enriched. For flow cytometry data, comparisons among three groups (CVic, SRic, and DMic) were performed using one-way analysis of variance (ANOVA) followed by Tukey’s *post hoc* test for multiple comparisons, or the Kruskal-Wallis test followed by Dunn’s test when the normality or homogeneity of variance assumptions were violated. Comparisons between two groups were conducted using the unpaired two-tailed Student’s *t* test (for normally distributed data) or the Mann-Whitney U test (for non-normally distributed data). Multiple testing corrections were also applied where applicable in flow cytometry comparisons. All statistical tests were two-sided, and a *P value* < 0.05 was considered statistically significant unless otherwise indicated. Data are presented as mean ± standard deviation (SD) or standard error of the mean (SEM) as specified in the figure legends.

## Results

### Single-cell landscape reveals distinct brain cell composition in mice infected with different viral strains

To determine an optimal sampling time point for scRNA-seq, we systematically monitored survival rate, body weight dynamics, clinical symptom scores, brain pathological alterations, serum neutralizing antibody levels, and bulk transcriptomic changes across consecutive time points (**Supplementary Figure S1**). The survival curve showed that mice in the CVS-11-infected (CVic) group began to die at 8–10 days post-infection (dpi) (**Supplementary Figure S1A**); marked body weight loss and progressively elevated clinical scores were observed in CVic mice over time (**Supplementary Figure S1B, C**). Histopathological examination revealed obvious brain tissue pathological lesions at 5 dpi between virulent and attenuated infection groups (**Supplementary Figure S1D**). Serum neutralizing antibody analysis indicated that SRV9-infected mice exhibited a prominent increase in neutralizing antibody levels exactly at 5 dpi (**Supplementary Figure S1E**). In addition, preliminary bulk RNA-seq results demonstrated that the number of differentially expressed genes peaked at 5 dpi (**Supplementary Figure S1F**). Collectively, 5 dpi was selected as the ideal time point for single-cell transcriptome profiling, as it precedes the onset of fatal mortality, coincides with obvious clinical and pathological changes, corresponds to the initial rise of adaptive humoral immunity, and represents the peak of global transcriptional perturbation in the brain.

**Figure 1:**
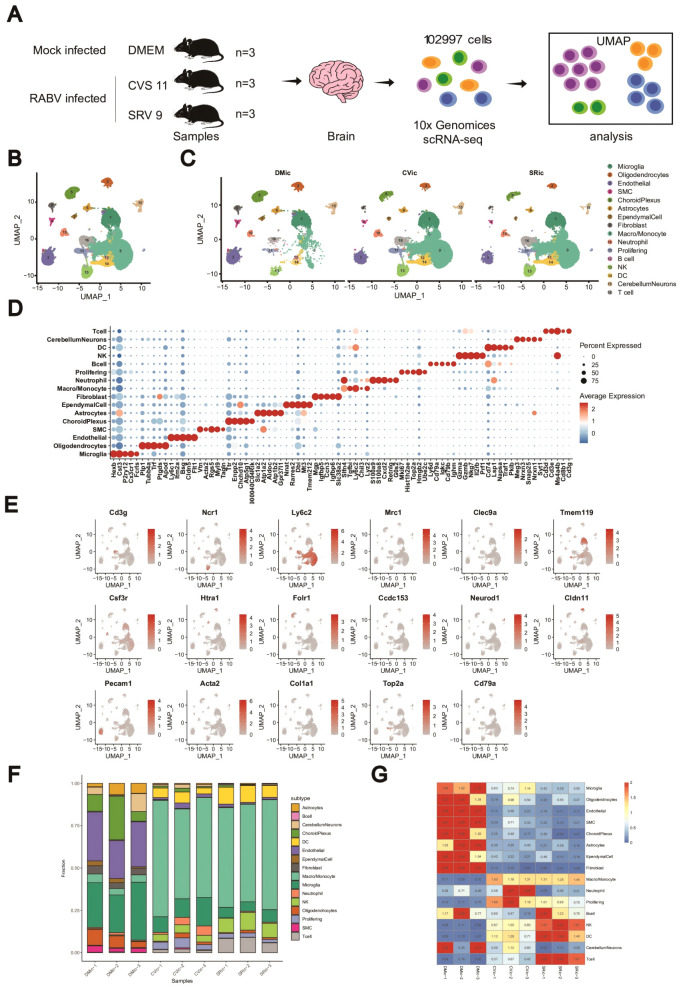
scRNA-seq shows cellular composition differences in mouse brains after RABV infection. **(A)** Schematic diagram showing the isolation of single cells from the brains of control and RABV-infected mice for scRNA-seq (n = 3 biologically independent mice per group). **(B)** Overview of cell types in the integrated single-cell transcriptome of 102,997 cells from the brains of control and RABV infected mice. **(C)** UMAP plot of cell types from mice with different disease stages. **(D)** Marker genes used to define each cell type; colored by gene expression in distinct cell types. **(E)** UMAP clustering and proportional distribution of different cell types in mice. **(F)** Proportions of different cell types in mice with different infection groups. **(G)** *R_o/e_* plot evaluating the relative enrichment or depletion of each major cell type across experimental groups (DMic, SRic, CVic); expected cell abundances were estimated via the chi-squared test.

Single-cell RNA sequencing was performed on brain tissues collected at 5 days post-infection (dpi) from CVS-11-inoculated (CVic), SRV9-inoculated (SRic), and mock-inoculated (DMic) mice, with three biological replicates per group. A total of 102,997 high-quality single cells were acquired for subsequent analysis (**Figure 1A**).

Unsupervised clustering and cell-type annotation identified 16 cell populations in mouse brain, including CNS resident cells (microglia, oligodendrocytes, astrocytes, cerebellar neurons, endothelial cells), myeloid and lymphoid immune cells (monocytes/macrophages, neutrophils, dendritic cells, NK cells, T cells, B cells), as well as stromal cells (fibroblasts, proliferating cells) (**Figures 1B–D**). Marker gene expression patterns confirmed the accuracy of cell annotation (**Figure 1E**).

**Figure 2:**
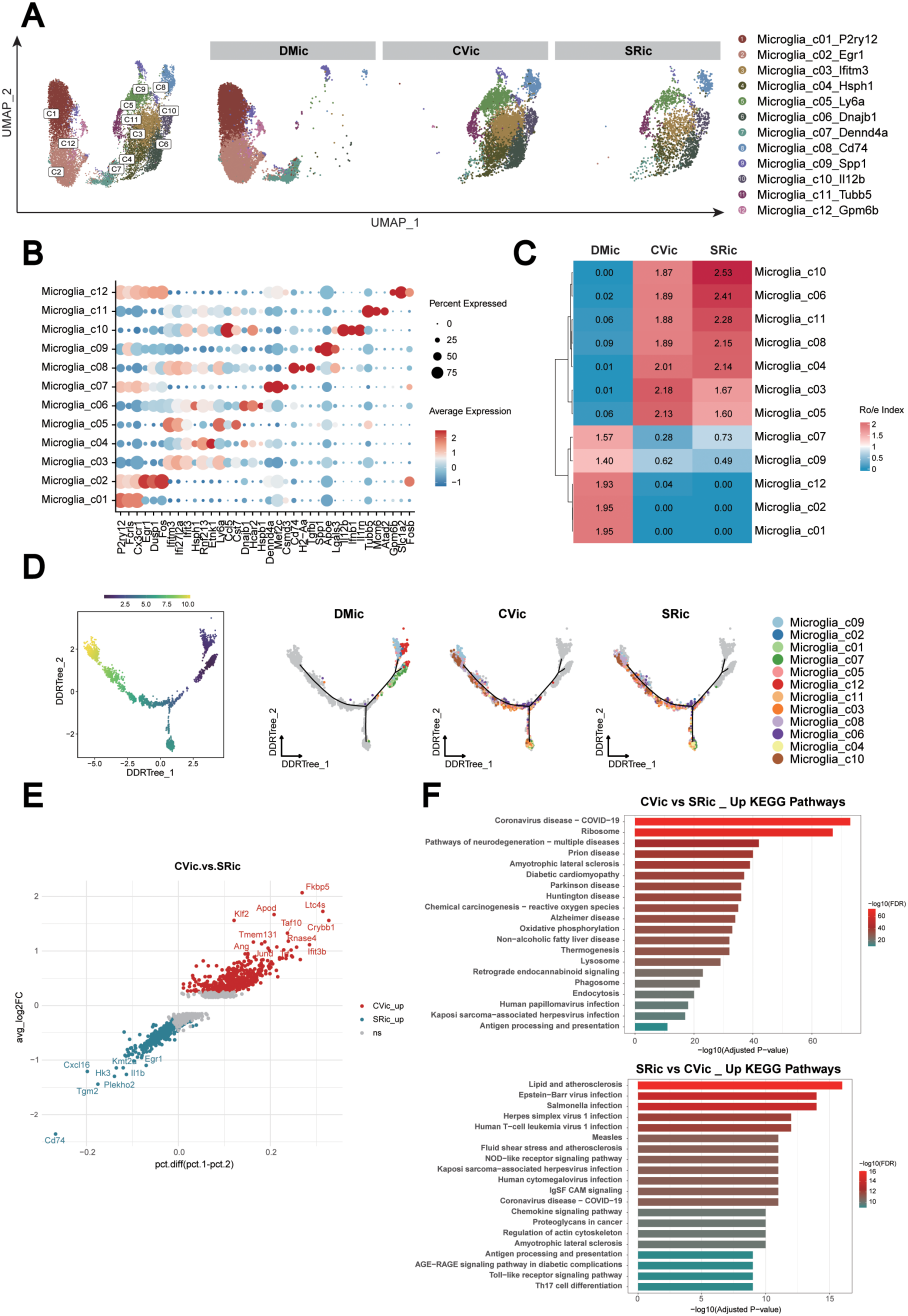
Microglial subset composition and transcriptional features in the brain after RABV infection. **(A)** UMAP plot showing the segregation of microglial subsets in the brains of control and RABV infected mice (n = 3 per group). **(B)** Marker genes used to define each cell subset. **(C)** *R_o/e_* plot evaluating the relative enrichment or depletion of microglial subsets across experimental groups (DMic, SRic, CVic); expected cell abundances were estimated via the chi-squared test. **(D)** Differentiation trajectories of brain microglia after infection with different RABV strains, reconstructed using Monocle 2 (DDRTree method). **(E)** Differentially expressed genes (DEGs) between CVic and SRic (upregulated in red, downregulated in blue), using Wilcoxon rank-sum test with Benjamini-Hochberg (BH) correction; adjusted *P* < 0.05 and |log_2_FC| > 0.25 were considered significant. **(F)** KEGG pathways enrichment analysis for DEGs between CVic and SRic microglia (BH-adjusted *P* < 0.05).

**Figure 3:**
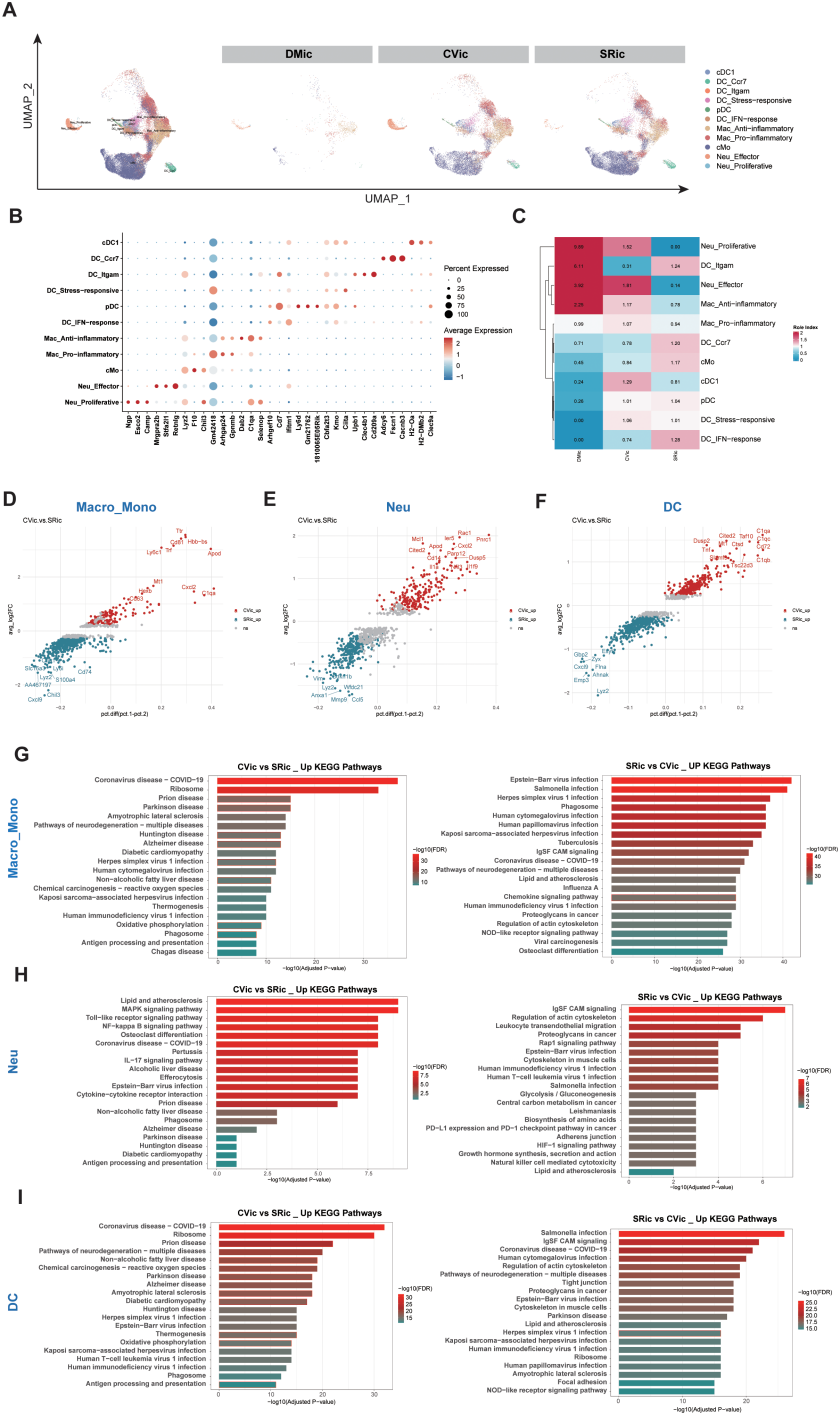
Myeloid cell subset composition and transcriptional profiles in the brain after RABV infection. **(A)** UMAP plot showing the segregation of myeloid cell subsets (DCs, neutrophils, macrophages, and monocytes) in the brains of control and RABV-infected mice (n = 3 per group). **(B)** Marker genes used to define each cell subset. **(C)** *R_o/e_* plot evaluating the relative enrichment or depletion of myeloid cell subsets across experimental groups (DMic, SRic, CVic); expected cell abundances were estimated via the chi-squared test. **(D–F)** Differentially expressed genes between CVic and SRic (upregulated in red, downregulated in blue). DEGs were identified using Wilcoxon rank-sum test with BH correction; adj. *P* < 0.05 and |log_2_FC| > 0.25. **(G–I)** KEGG pathways for CVic vs SRic. All KEGG analyses used hypergeometric test with BH correction (adj. *P* 0.05).

**Figure 4:**
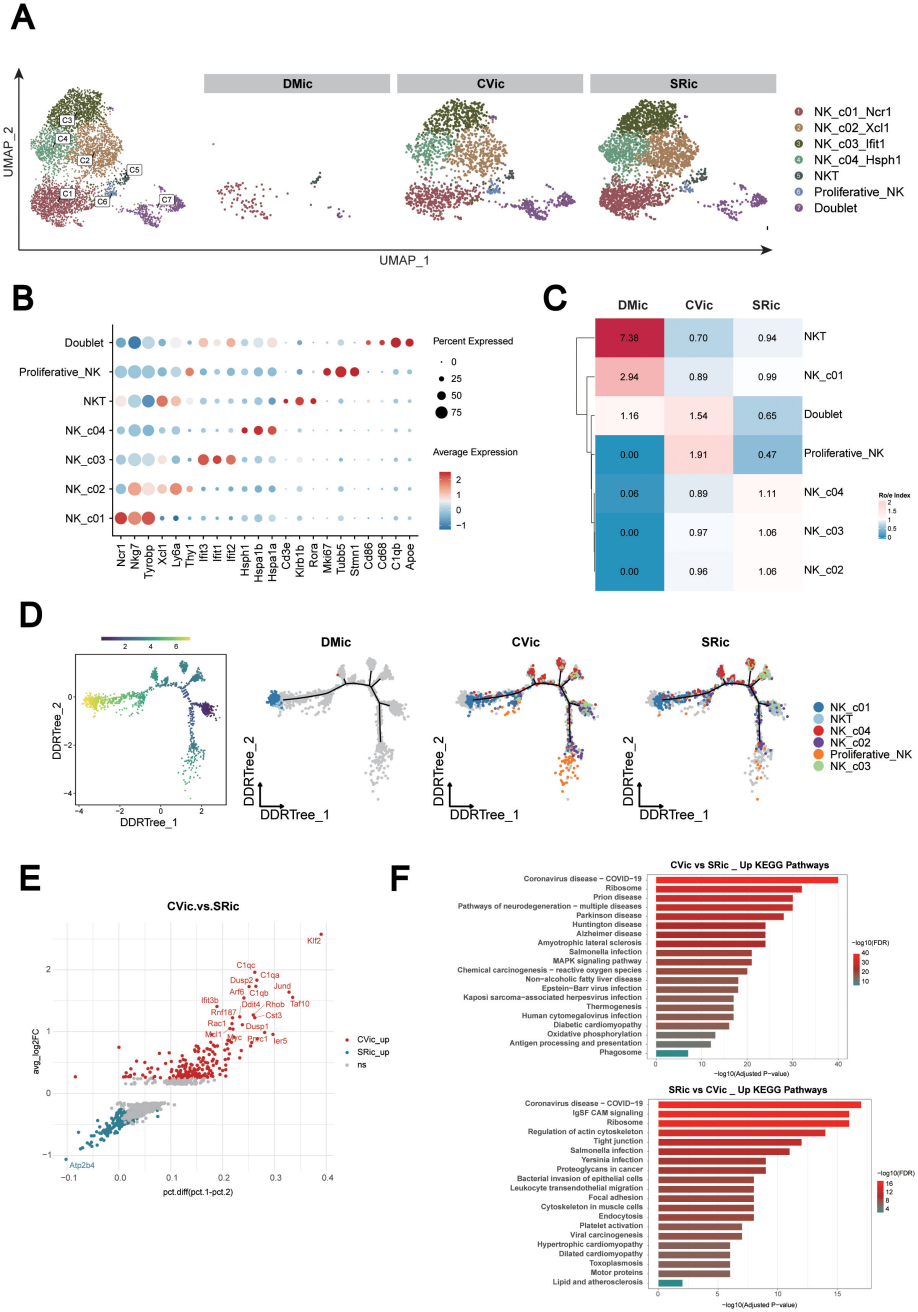
NK cell subset composition and functional features in the brain after RABV infection. **(A)** UMAP plot showing the segregation of NK cell subsets in the brains of control and RABV infected mice (n = 3 per group). **(B)** Marker genes used to define each cell subset. **(C)** *R_o/e_* plot evaluating the relative enrichment or depletion of NK cell subsets across experimental groups (DMic, SRic, CVic); expected cell abundances were estimated via the chi-squared test. **(D)** Differentiation trajectories of brain NK cells after infection with different RABV strains, reconstructed using Monocle 2 (DDRTree). **(E)** Differentially expressed genes between CVic and SRic (upregulated in red, downregulated in blue). DEGs: Wilcoxon rank-sum test with BH correction, adj. *P* < 0.05, |log_2_FC| > 0.25. **(F)** KEGG pathway enrichment for DEGs between CVic and SRic NK cells (hypergeometric test with BH correction, adj. *P* < 0.05).

**Figure 5:**
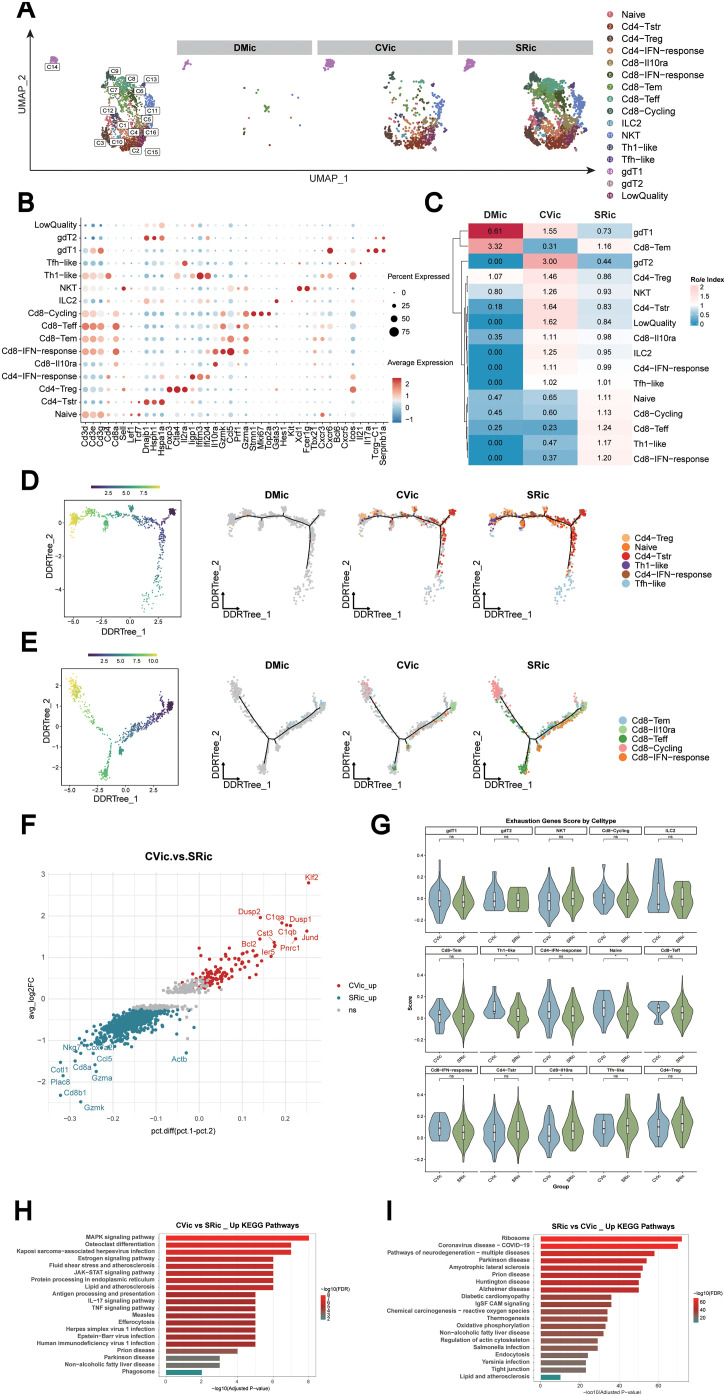
T cell subset composition, differentiation, and exhaustion associated features in the brain after RABV infection. **(A)** UMAP plot showing the segregation of T cell subsets in the brains of control and RABV infected mice (n = 3 per group). **(B)** Marker genes used to define each cell subset. **(C)** *R_o/e_* plot evaluating the relative enrichment or depletion of T cell subsets across experimental groups (DMic, SRic, CVic); expected cell abundances were estimated via the chi-squared test. **(D)** Differentiation trajectories of brain CD4+ T cells after infection with different RABV strains (Monocle 2, DDRTree). **(E)** Differentiation trajectories of brain CD8+ T cells after infection with different RABV strains (Monocle 2, DDRTree). **(F)** Differentially expressed genes between CVic and SRic (upregulated in red, downregulated in blue). DEGs: Wilcoxon rank-sum test with BH correction, adj.P.Val < 0.05, |log_2_FC| > 0.25. **(G)** T cell exhaustion scores under different RABV infections.Statistical significance indicators: ns, not significant (*P*≥0.05);*P < 0.05. All tests were two-sided. **(H, I)** KEGG pathway enrichment for DEGs between CVic and SRic T cells (hypergeometric test with BH correction, adj.P.Val < 0.05).

Cell proportion analysis showed obvious strain-specific alterations in brain immune cell composition. Compared with the DMic control group, both CVic and SRic infections increased the proportions of monocytes/macrophages and neutrophils, with comparable levels between the two infected groups. The SRic group showed expanded myeloid cells predominantly driven by increased dendritic cell proportions. Notably, monocyte/macrophage proportions were markedly higher in both infected groups relative to the DMic control. In lymphoid populations, T and NK cell proportions were elevated in both CVic and SRic groups compared with the DMic control, although the increases were more pronounced in the SRic group than in the CVic group. The proportions of astrocytes and oligodendrocytes exhibited a moderate reduction in both CVic and SRic groups compared with the DMic control, without reaching statistically significant differences (**Figure 1F**). Of note, the *R_o/e_* plot for global cell types (**Figure 1G**) displays individual mice to show biological variability, whereas subsequent *R_o/e_* plots for immune subsets (**Figure 2C**-**5C**) present group−pooled data to highlight infection−driven compositional shifts.

Global gene expression profiles showed obvious differences among CVic, SRic and DMic groups (**Supplementary Figure S2A–C**). GO and KEGG enrichment displayed distinct functional enrichment patterns between virulent and attenuated strain infection groups (**Supplementary Figure S2D–O**).

### Microglial phenotypes differ between pathological and protective immune responses

Unsupervised subclustering divided microglia into 12 functional subsets with specific marker gene profiles (**Figures 2A, B**). UMAP (Uniform Manifold Approximation and Projection) visualization presented distinct subset distribution patterns between CVic and SRic groups.

Subset proportion analysis revealed strain-specific remodeling of microglia. In the CVic group, homeostatic microglia (*P2ry12*+, *Tmem119*+) and neuroprotective subsets (*Gpm6b*+, *Bdnf*+) were decreased, whereas proinflammatory subsets (*Il1b*+, *Tnf*+, *Nlrp3*+), interferon-responsive subsets (*Ifitm3*+, *Isg15*+) and proliferative subsets (*Dnajb1*+, *Top2a*+, *Mki67*+) were increased. In the SRic group, homeostatic microglia were reduced, while antigen-presenting subsets (*Cd74*+, *H2-Aa*+, *H2-Eb1*+), tissue repair subsets (*Spp1*+, *Tgm2*+, *Il10*+) and chemotactic subsets (*Ccl2*+, *Ccl5*+, *Cxcl9*+) were elevated, without obvious expansion of proinflammatory populations (**Figure 2C**).

Pseudotime trajectory analysis revealed that microglia in the CVic group predominantly showed transcriptional transitions from homeostatic and neuroprotective subsets toward subsets expressing pro-inflammatory and phagocytic markers. Microglia in the SRic group displayed transcriptional trajectory patterns consistent with a shift from homeostatic subsets toward antigen-presenting, tissue repair and chemotactic molecular signatures (**Figure 2D**).

Differential gene expression analysis identified distinct transcriptional signatures. Genes including *Fkbp5, Ltc4s, Apod* and Jund were specifically upregulated in CVic microglia, together with elevated expression of ribosomal protein, lysosomal enzyme and mitochondrial respiratory chain-related genes. In SRic microglia, upregulated genes were enriched in antigen presentation, tissue repair, phagocytosis, and immune activation-related molecules such as *Cd74, Tgm2, Plekho2* and *Kmt2a*, accompanied by increased expression of pro-inflammatory factors, chemokines and interferon-stimulated genes (**Figure 2E**).

KEGG pathway enrichment showed different functional patterns. The CVic group was enriched in Ribosome, Oxidative phosphorylation, Lysosome, and multiple neurodegenerative disease-related pathways. The SRic group was enriched in Antigen processing and presentation, Toll-like receptor signaling, Chemokine signaling and actin cytoskeleton regulation pathways (**Figure 2F**).

### Two RABV strains drive distinct transcriptional profiles of myeloid cell subsets in mouse brains

Brain-infiltrating myeloid cells were classified into 11 subsets, including 6 dendritic cell subsets, 3 monocyte/macrophage subsets and 2 neutrophil subsets (**Figure 3A**).

Subset proportion analysis showed evident differences between groups. The CVic group had increased proliferative neutrophils, effector neutrophils, pro-inflammatory/anti-inflammatory macrophages and cDC1. The SRic group was dominated by *Itgam*+ dendritic cells, *Ccr7*+ dendritic cells, classical monocytes and interferon-responsive dendritic cells (**Figures 3B, C**).

Distinct DEG profiles were observed in myeloid cells of two infection groups. Genes related to inflammation, stress response and anti-apoptosis (*Nfil3, Parp12, Cd14, Il1a, Il1f9, Mcl1, Cd81*) were upregulated in CVic myeloid cells. Genes involved in antigen presentation, chemotaxis, and tissue remodeling (*Ccl5, Lyz2, Vim, Anxa1, Mmp9, Cd74, Cxcl9*) were increased in SRic myeloid cells (**Figures 3D–F**).

Pathway enrichment showed lineage-specific differences. In CVic dendritic cells and monocytes/macrophages, pathways related to ribosome biogenesis, oxidative phosphorylation, lysosome dysfunction and MHC I antigen presentation were enriched (**Figure 3G**). CVic neutrophils showed strong activation of Toll-like receptor, NOD-like receptor, apoptosis and necroptosis pathways (**Figure 3H**). In the SRic group, monocytes/macrophages were enriched in phagocytosis, antigen presentation and chemokine signaling pathways; dendritic cells were enriched in actin cytoskeleton and immune receptor signaling; neutrophils showed elevated leukocyte migration and glycolysis pathway activity (**Figures 3H, I**).

### NK cells exhibit altered functional profiles after virulent RABV infection

NK cells were clustered into six functional subsets plus one doublet population (NK_c01_Ncr1, NK_c02_Xcl1, NK_c03_Ifit1, NK_c04_Hsph1, NKT, Proliferative_NK) (**Figure 4A**). Marker gene expression confirmed subset specificity (**Figure 4B**).

Subset proportion analysis indicated obvious strain-specific changes. The proliferative NK subset was significantly expanded in the CVic group, while other functional subsets were decreased. NK_c02_Xcl1, NK_c03_Ifit1 and NK_c04_Hsph1 subsets were elevated in the SRic group (**Figure 4C**).

Pseudotime analysis suggested that NK cells in the CVic group transcriptionally converged toward a proliferative subset, whereas those in the SRic group showed trajectory patterns consistent with antiviral gene expression signatures (**Figure 4D**).

DEG analysis revealed unique transcriptional features. CVic NK cells upregulated *Klf2*, *Jund, Taf10*, complement components, *Dusp1/2, Rhob, Cst3, Ddit4, Ifit3b, Myc* and *Mcl1*, without obvious elevation of cytotoxicity-related genes. SRic NK cells showed increased expression of *Atp2b4* and multiple antiviral and immune activation-related genes (**Figure 4E**).

KEGG enrichment presented distinct pathway patterns. The CVic group was enriched in neurodegenerative disease, metabolism, apoptosis, MAPK and inflammatory signaling pathways. The SRic group was enriched in cytoskeleton regulation, cell adhesion, leukocyte migration and natural killer cell cytotoxicity pathways (**Figure 4F**).

### T cell exhaustion and differentiation features are observed in lethal infection

T cells were divided into 15 subsets plus one low-quality population (Naive, Cd4-Tstr, Cd4-Treg, Cd4-IFN-response, Tfh-like, Th1-like, Cd8-Il10ra, Cd8-IFN-response, Cd8-Tem, Cd8-Teff, Cd8-Cycling, ILC2, NKT, gdT1, gdT2) (**Figure 5A**). Marker gene expression verified subset annotation (**Figure 5B**).

Subset proportions differed significantly between groups. Regulatory T cell subsets (Cd4-Treg, Cd8-Il10ra) were increased while effector T cell subsets were reduced in the CVic group. Effector T cells and gdT2 subsets were elevated in the CVic group (**Figure 5C**).

Pseudotime trajectory analysis suggested that T cells in the CVic group exhibited less pronounced transcriptional progression toward effector and memory gene expression programs, whereas those in the SRic group showed trajectory patterns more consistent with effector and transcriptional progression (**Figures 5D, E**).

T cell exhaustion score analysis showed that Th1-like and naive T cells had high exhaustion scores in the CVic group; the Cd8-Il10ra subset displayed elevated exhaustion scores in the SRic group (**Figure 5G**).

DEG patterns were distinct between groups. Inhibitory and anti-apoptotic genes (*Klf2, Dusp1, Dusp2, Socs3, Tsc22d3, Bcl2, Mcl1*) and complement genes were upregulated in CVic T cells. Cytotoxic, chemokine, migration and T cell activation-related genes were increased in SRic T cells (**Figure 5F**).

KEGG enrichment showed that the CVic group was enriched in osteoclast differentiation, JAK-STAT, MAPK, inflammatory signaling and endoplasmic reticulum processing pathways. The SRic group was enriched in ribosome metabolism, cytoskeleton regulation, leukocyte migration, T cell receptor signaling, Th1/Th2 differentiation and cytotoxicity pathways (**Figures 5H, I**).

### Intercellular communication patterns differ in the CNS following RABV infection

**Figure 6:**
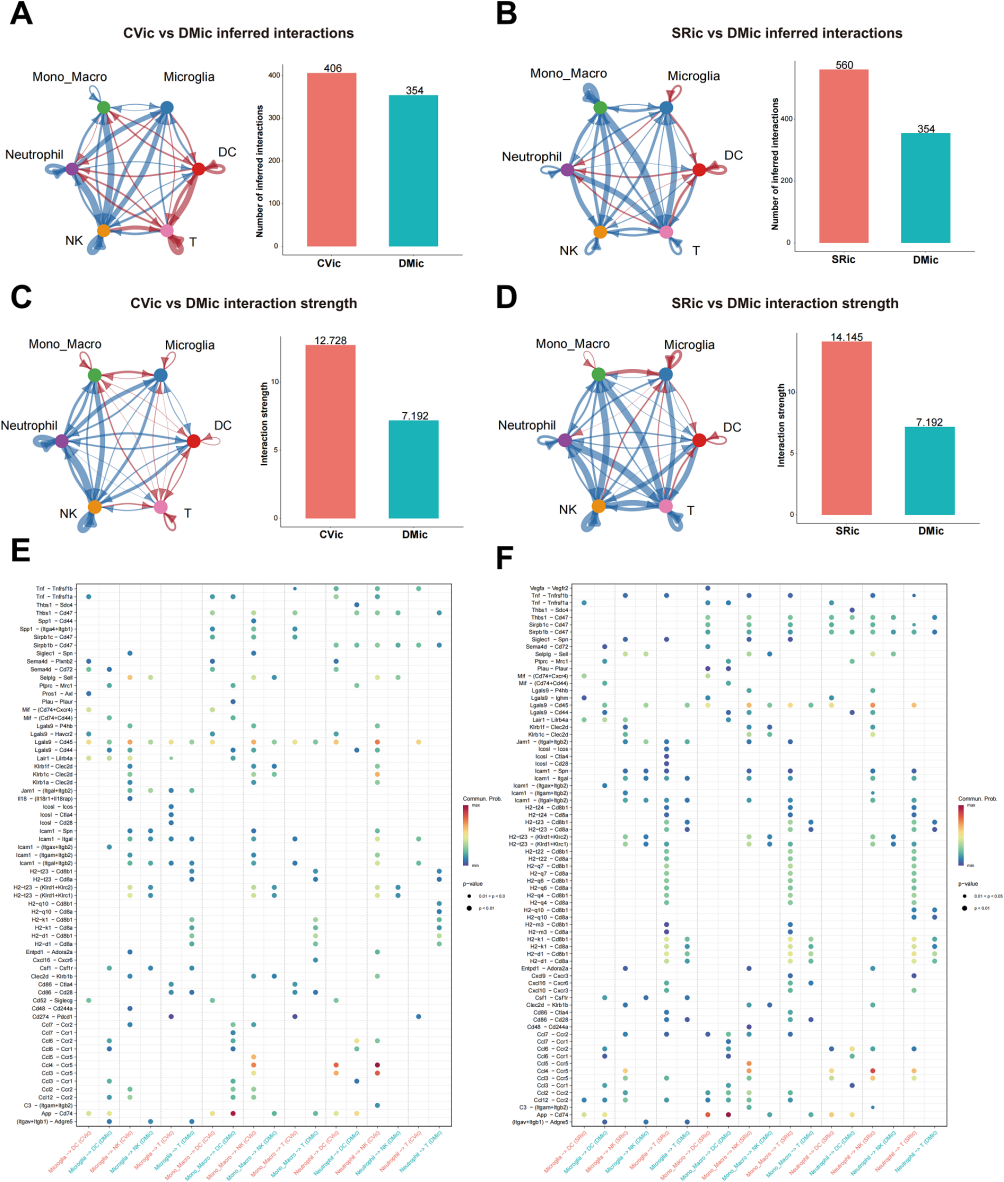
Altered intercellular communication in the mouse brain after RABV infection. **(A–D)** Circle plots showing the number **(A, B)** and strength **(C, D)** of interactions among different brain cell types. Line thickness indicates the quantity and strength of signals. Red lines represent increased interactions, and blue lines represent decreased interactions. Histograms show the number and strength of interactions. Cell-cell communication was inferred using CellChat (v1.1.0) based on ligand-receptor expression. **(E)** Ligand–receptor pairs between immune cells in CVic vs DMic. **(F)** Ligand–receptor pairs between immune cells in SRic vs DMic. Statistical significance of interaction probability was assessed using CellChat’s permutation test (n = 100 permutations, *P* < 0.05).

Cell–cell communication analysis showed obvious differences in interaction number and strength among three groups. The CVic group tended to display inferred reduced quantity and intensity of cell–cell communication, while the SRic group exhibited predicted strengthened intercellular crosstalk with dense signaling networks (**Figures 6A–D**). Ligand-receptor pair analysis indicated inferred weakened antigen presentation, chemotaxis and costimulatory signaling in the CVic group, alongside relative enrichment of predicted inhibitory signaling pathways. By contrast, the SRic group showed enriched transcriptomically predicted ligand–receptor pairs linked to antigen processing, chemokine axis, cell adhesion, immune synapse formation, T cell activation and NK cell cytotoxicity (**Figures 6E, F**).

### Flow cytometry confirms distinct CNS immune remodeling

**Figure 7:**
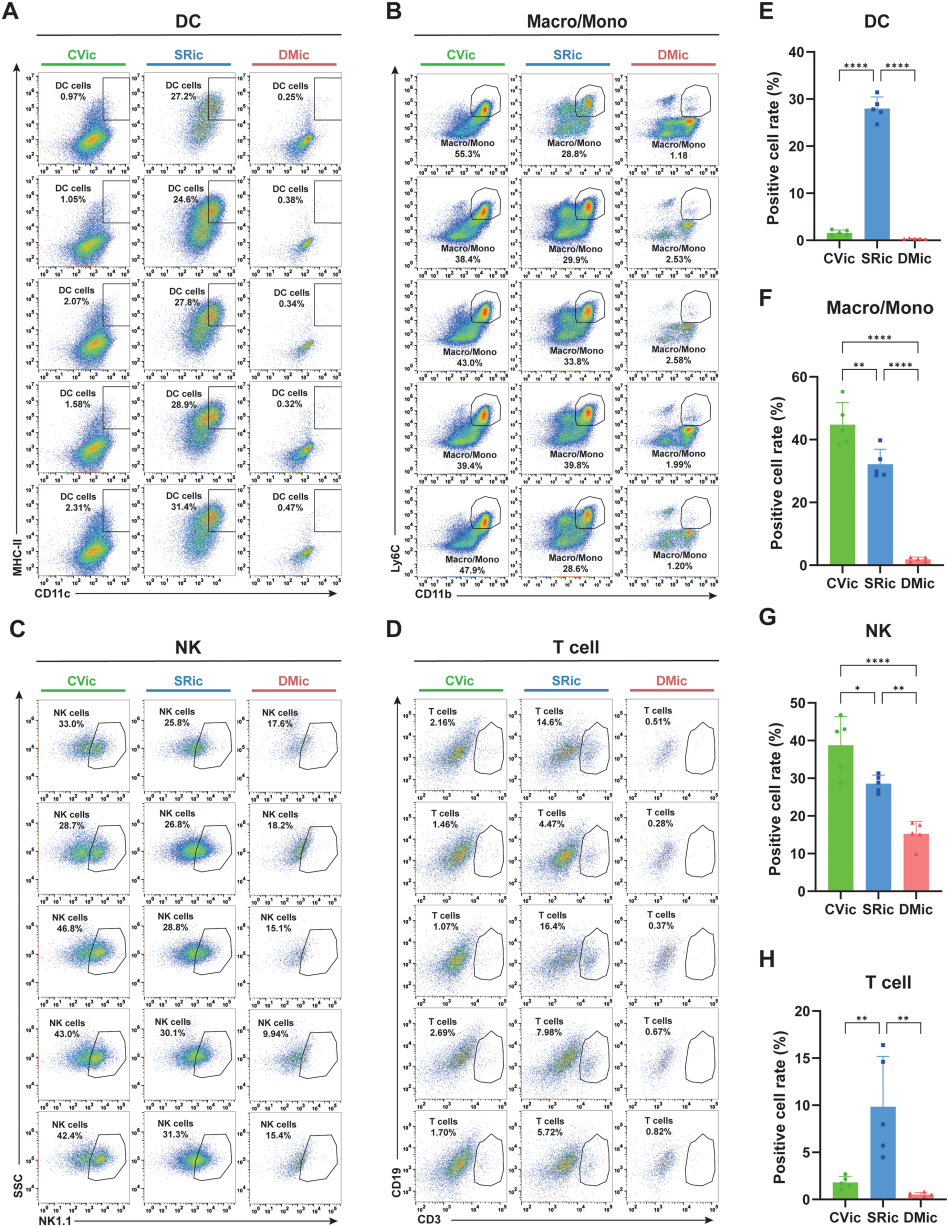
Flow cytometric analysis of immune cell subsets in mouse brain at 5 dpi. **(A)** Representative flow plots and proportional statistics of DCs in DMic, SRic and CVic groups. **(B)** Representative flow plots and proportion analysis of monocyte/macrophage populations among three groups. **(C)** Flow cytometric profiles and quantitative distribution of NK cells in different infection groups. **(D)** Representative flow diagrams and proportion statistics of T cells in the three experimental groups. **(E)** Quantification of the percentage of DCs. **(F)** Quantification of the percentage of monocyte/macrophage. **(G)** Quantification of the percentage of NK cells. **(H)** Quantification of the percentage of T cells. Statistical significance was assessed using one-way ANOVA (*P < 0.05, **P < 0.01, ***P < 0.001, and ****P < 0.0001).

To further validate the composition and functional activation of immune subpopulations in the central nervous system, we performed flow cytometric analysis on brain tissue harvested from five mice per group at 5 days post-infection. The detailed flow cytometric gating strategy for identifying brain immune cell subsets is shown in **Supplementary Figure S3**. Quantification of total leukocytes, monocyte/macrophages, dendritic cells (DCs), NK cells, and T cells showed that, relative to the DMic control group, the CVic (virulent strain) group exhibited a marked increase in total *CD45*+ leukocytes in brain tissue, indicating robust immune cell infiltration (**Figures 7A–D**). Strikingly, however, this group displayed significantly reduced proportions of functional NK cells and *CD3*+ T cells, along with an elevated proportion of *CD11b*+ *Ly6C*+ monocyte/macrophages and a decreased proportion of *CD11c*+ *MHC-II*+ mature DCs. Quantitative analysis confirmed that the percentages of *CD11c*+ *MHC-II*+ DCs (**Figure 7E**), *CD11b*+ *Ly6C*+ monocytes/macrophages (**Figure 7F**), *NK1.1*+ NK cells (**Figure 7G**), and *CD3*+ T cells (**Figure 7H**) were significantly altered between groups. In sharp contrast, the SRic (attenuated strain) group showed substantially increased proportions of *CD11c*+ *MHC-II*+ DCs, NK cells, and *CD3*+ T cells, consistent with a profile of effective antiviral immune activation and coordinated immune cell recruitment. These flow cytometric results are highly consistent with the immune landscape identified by single-cell RNA sequencing, which revealed immune dysfunction and exhaustion in the CVic group versus robust protective immune activation in the SRic group. Together, these independent validation data strongly confirm the divergent patterns of CNS immune remodeling induced by virulent and attenuated RABV strains.

## Discussion

Single-cell RNA sequencing provides an unprecedented resolution for dissecting the heterogeneous responses of immune cells within the CNS following viral infection. In this study, we performed scRNA−seq to systematically compare the CNS immune landscape of mice infected with the virulent CVS−11 strain versus the attenuated SRV9 strain. By profiling over 100,000 cells, we have constructed a high−resolution atlas that reveals, for the first time, cell−subset−specific transcriptional signatures associated with lethal versus protective outcomes of rabies virus infection. This comparative design thus represents a novel approach to unraveling the cellular and molecular basis underlying rabies pathogenesis.

In accordance with previous studies demonstrating that rabies virus infection modulates microglial activation, our scRNA-seq analysis refined this concept by identifying 12 distinct microglial subsets. Notably, CVS-11 infection drove microglia away from homeostatic and neuroprotective subsets toward pro-inflammatory and pathological phagocytic subsets, whereas SRV9 infection promoted differentiation toward antigen-presenting, tissue repair and chemotactic subsets. Moreover, we observed that CVS-11 infected microglia specifically upregulated *Apod* and *Fkbp5*. Zhang et al. reported that Apolipoprotein D (*Apod*) facilitates rabies virus propagation by interacting with the viral G protein and upregulating cholesterol. However, beyond its proviral effect, ApoD has also been implicated as a stress−induced lipocalin upregulated in the nervous system following injury or neurodegenerative pathologies, functioning as an evolutionarily conserved anti−stress and anti−inflammatory molecule through the activation of phospholipase A2 signaling pathways and the inhibition of Toll−like receptors to mitigate excessive inflammation (23, 24). *Fkbp5*, a stress−inducible gene encoding a negative cochaperone of the glucocorticoid receptor, has emerged as a critical regulator of microglial activation and neuroinflammation. Recent studies demonstrate that *Fkbp5* facilitates the assembly of the IκB kinase complex for NF−κB activation and that its genetic ablation or pharmacological inhibition attenuates microglial activation and suppresses interferon−gamma−induced inflammatory programs (25, 26). Furthermore, *Fkbp5* has been implicated in systemic inflammation−induced neuroinflammation and hippocampal glucocorticoid receptor activation, contributing to inflammation−induced anxiety (27). Taken together, the upregulation of *Fkbp5* in CVS−11−infected microglia may therefore represent a transcriptional signature linking pathological neuroinflammation to the lethal outcome of rabies virus infection. Our transcriptomic data raise the possibility that divergent activation patterns of microglia, suggestive of pathological or immunoregulatory phenotypes, may contribute to differential infection outcomes, but this interpretation requires functional validation.

Our analysis of brain infiltrating myeloid cells revealed that CVS-11 infection was characterized by expansion of proliferative and effector neutrophils, together with strong activation of TLR/NLR inflammatory signaling pathways. In contrast, SRV9 infection promoted antigen presentation, chemokine signaling and leukocyte migration pathways. Early work by Lafon established that rabies virus employs multiple subversive strategies to evade host immunity, including the induction of T cell apoptosis and the expression of immunosuppressive molecules in the CNS (28). The excessive neutrophilic inflammation observed in CVS-11 infected mice is consistent with the concept that virulent strains drive a myeloid response that contributes to tissue injury rather than effective viral control. The upregulation of *Il1a* and *Cd14* in CVS-11 infected myeloid cells further supports the presence of a pro-inflammatory, dysregulated state (29, 30). Conversely, the enrichment of *Ccl5*, *Cd74* and *Cxcl9* in SRV9-infected myeloid cells suggests that attenuated strains promote coordinated, chemokine-driven recruitment of antigen-presenting cells and T cells, which is essential for viral clearance from the CNS (31–33).

In CVS-11 infected mice, NK cells exhibited a striking expansion of the proliferative subset, accompanied by upregulation of *Klf2*, *Dusp1*, and *Dusp2*. Recent studies have highlighted that during chronic viral infection, high expression of *Klf2* in T cells suppresses effector differentiation and promotes exhaustion-associated transcription programs (34, 35). Although our knowledge of KLF2 function in NK cells is less established, the upregulation of *Klf2* in CVic NK cells, together with the lack of corresponding upregulation of cytotoxicity-related genes (*Gzmb*, *Prf1*), suggests that these cells may adopt a proliferative but functionally blunted state (36, 37). This pattern is consistent with the hypothesis that virulent RABV strains may impair NK cell-mediated antiviral activity, although direct evidence of functional impairment is lacking, but a concept supported by earlier observations that pathogenic RABV infection subverts innate immune clearance. In contrast, SRV9 infection directed NK cell differentiation toward subsets enriched in antiviral and cytotoxic pathways, indicating that attenuated strains can effectively harness NK cell functions for CNS defense.

Our analysis of T cell subsets revealed that CVS-11 infection was marked by increased regulatory T cell proportions, reduced effector T cell subsets, and elevated exhaustion scores in Th1-like and naive T cells. Mechanistically, T cells from CVS-11 infected mice upregulated *Klf2*, *Socs3*, *Dusp1*, and *Dusp2*. The role of *Klf2* in driving early T cell exhaustion has been firmly established: *Klf2* programs early exhausted T cell states and restrains antiviral immunity during chronic viral infection (34). Similarly, *Socs3*, a potent suppressor of cytokine signaling, impairs T cell function and promotes T cell exhaustion, thereby contributing to viral persistence (38). The concurrent upregulation of *Klf2* and *Socs3* in CVic T cells therefore likely represents a key transcriptional signature driving T cell dysfunction in lethal rabies. In contrast, SRV9 infection promoted enrichment of effector and memory T cell subsets, with activation of T cell receptor signaling and Th1/Th2 differentiation pathways. These findings are consistent with the well-established concept that rabies virus eliminates protective T cells in the CNS via PD-1/PD-L1-dependent mechanisms.

Several limitations of this study must be acknowledged. First, our analysis was limited to a single time point (5 days post-infection); although this time point was carefully selected based on preliminary data, the absence of a time series restricts inferences about the dynamic evolution of immune responses. Second, the use of intracerebral inoculation does not recapitulate the natural peripheral route of rabies infection, and the immune responses in this model may therefore differ from naturally acquired rabies. Third, the sample size (n = 3 per group) and the relatively lenient DEG threshold (|log2FC| > 0.25, *P* < 0.05) may limit statistical power and include potential false positives, although the latter is widely accepted in scRNA-seq analyses to capture subtle biological signals. Fourth, as a transcriptome-only study, all interpretations of cellular function remain predictive and require functional validation. Importantly, we plan to perform qPCR, Western blot and immunohistochemistry in follow-up experiments to directly confirm key findings, including the expression levels of *Fkbp5*, *Apod*, *Klf2* and *Socs3* across immune subsets, as well as protein markers of T cell exhaustion (PD-1, TIM-3) and NK cell cytotoxicity (GZMB, PRF1). Such orthogonal validation will substantially strengthen the reliability of the conclusions drawn from scRNA-seq data. Finally, our study was conducted exclusively in female C57BL/6 mice; whether these findings are generalizable to males or to other mouse strains remains to be determined.

Through single-cell transcriptomic profiling, we observed that the transcriptional landscapes distinguishing lethal and attenuated RABV infections are defined by cell-type-specific programmes rather than uniform tissue-wide shifts. In contrast to conventional models of generalized microglial activation, our data reveal that virulent CVS-11 infection is associated with a transcriptional signature indicative of a stress-phagocytic axis (*Apod, Fkbp5*), whereas attenuated SRV9 correlates with an immunoregulatory profile (*Cd74, Spp1*). These findings provide a cellular context for previously reported NK cell dysfunction and T-cell impairment, highlighting distinct transcriptional states: an NK cell subset characterized by high proliferative marker expression coupled with low cytotoxic effector gene signatures, and a convergence of diverse T-cell subsets toward a shared inhibitory transcriptional state(*Klf2, Socs3, Dusp1*) (14).

In summary, this study presents a high-resolution single-cell transcriptomic atlas profiling CNS immune responses following infection with virulent and attenuated rabies virus strains. Notably, our work represents an early application of scRNA-seq to dissect the central immune landscape during RABV infection. Based solely on transcriptomic profiling, our findings tentatively suggest that lethal CVS-11 infection may be accompanied by pathological microglial polarization, exaggerated neutrophilic inflammation, proliferative but functionally compromised NK cell phenotypes, and potential T cell exhaustion possibly linked to the upregulation of *Klf2* and *Socs3*. By comparison, the attenuated SRV9 strain appears to favor antigen-presenting microglial subpopulations, coordinated myeloid cell responses, robust NK cell activation, and competent effector T cell differentiation. The predicted signature genes *Fkbp5, Apod, Klf2*, and *Socs3* associated with lethal RABV infection may serve as promising molecular candidates for further experimental validation and future therapeutic development. Collectively, these single-cell data provide a predictive framework for understanding rabies immunopathogenesis and offer a valuable transcriptomic resource to inform future rational vaccine design and immunotherapeutic strategies.

## Data Availability

All raw sequencing data have been uploaded to the NCBI’s Gene Expression Omnibus database (BioProject: https://www.ncbi.nlm.nih.gov/search/all/?term=PRJNA1447729). Any additional information required to reanalyze the data reported in this paper is available from the lead contact upon request. The experimental code related to this article is open source and available on GitHub at: https://github.com/WangXinyue97/rabies-scrna-analysis.git.

## Supplementary material

Supplementary Figure 1 Time-point validation and comprehensive baseline characteristics of RABV-infected mice. **(A)** Survival curves of mice in CVic, SRic and DMic groups within 10 dpi (n= 10 per group). **(B)** Body weight changes in control and RABV infected mice (n= 3 per group). **(C)** Clinical signs were scored on a scale from 0 to 5 (n= 4 per group). **(D)** Histopathology of mouse brains after intracerebral infection with RABV. Green arrows indicate vascular cuffs; red arrows indicate neuronal necrosis; yellow arrows indicate gliosis. **(E)** Dynamic changes of serum neutralizing antibody levels in RABV-infected mice (n= 3 per group). **(F)** Number of differentially expressed genes at different time points based on bulk RNA-seq.

Supplementary Figure 2 Global gene expression changes in mouse brains after RABV infection. **(A)** Top 10 differentially expressed genes in each group. **(B)** Overlap of upregulated and downregulated differentially expressed genes among different strains. **(C)** Volcano plots of upregulated and downregulated differentially expressed genes for different strains. **(D–I)** GO pathway analysis of upregulated and downregulated differentially expressed genes for different strains. (Yellow: Biological Process; Red: Cellular Component; Purple: Molecular Function.). **(J-O)** KEGG pathway analysis of upregulated and downregulated differentially expressed genes for different strains. DEGs were defined as those with adjusted P < 0.05 and |log_2_ fold change| > 0.25. GO and KEGG enrichment analyses were performed using clusterProfiler (version 4.2.2) with hypergeometric test and BH correction; terms with adjusted *P* < 0.05 were considered statistically significant.

Supplementary Figure 3 Flow cytometric gating strategy for immune cell subsets in mouse brain. **(A)** Sequential gating workflow for identifying single live CD45+ leukocytes, followed by the separation of *CD11b*^+^ myeloid subsets (monocytes/macrophages, DCs) and NK cells based on the expression of *CD11b, Ly6C, CD11c* and *MHC-II*. **(B)** Gating strategy for sorting CD3+ T cells and *CD19*+ B cells within lymphocyte populations.
