# Supplementary figures and images for: Single-cell immune landscape of the central nervous system of mice infected with rabies virus
Source: Front Immunol. 2026 Jul 2;17:1850356. doi: 10.3389/fimmu.2026.1850356 (PMC13373092; doi:10.3389/fimmu.2026.1850356)

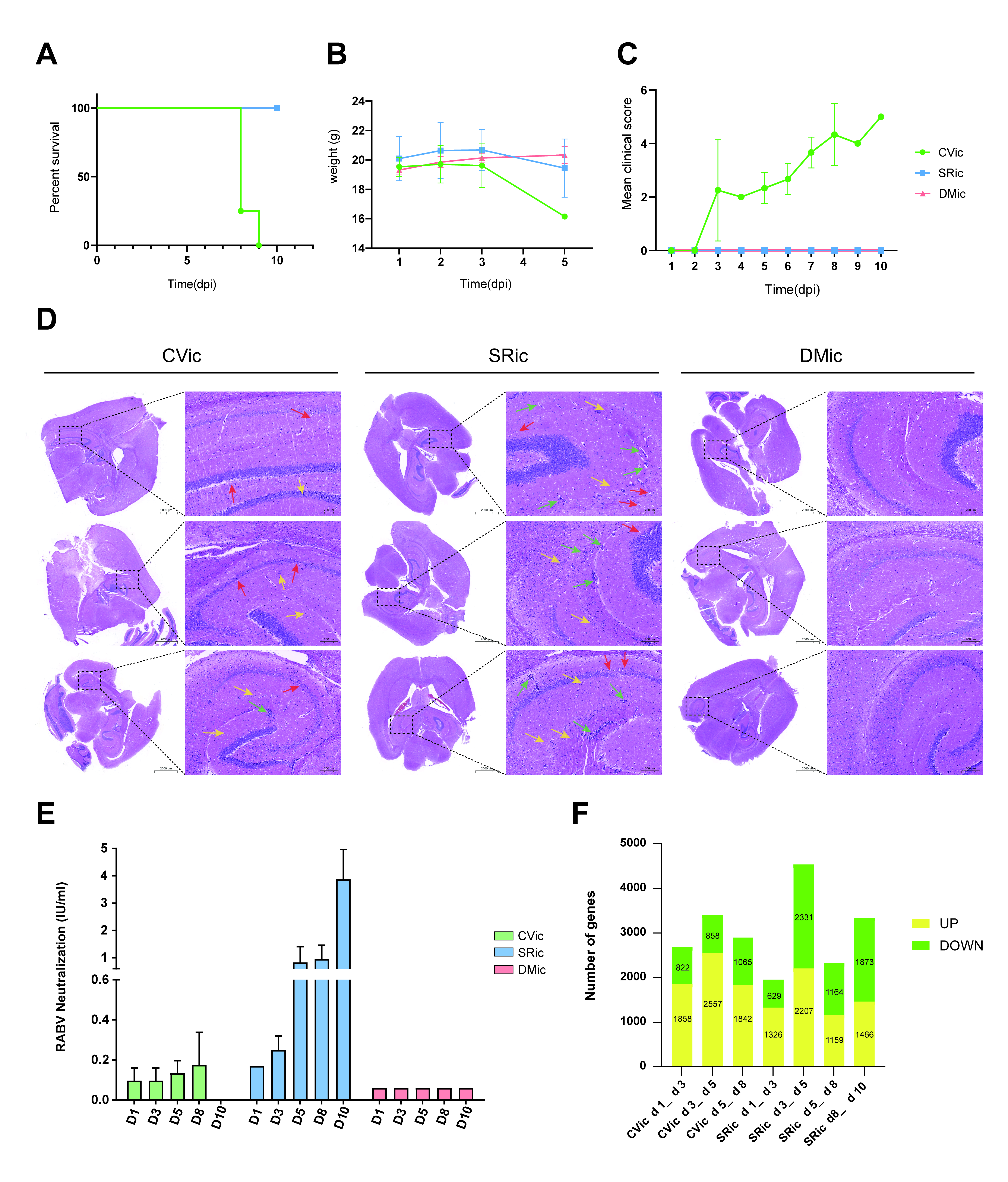

Supplement: Supplementary Figure 1 — Time-point validation and comprehensive baseline characteristics of RABV-infected mice. (A) Survival curves of mice in CVic, SRic and DMic groups within 10 dpi (n= 10 per group). (B) Body weight changes in control and RABV infected mice (n= 3 per group). (C) Clinical signs were scored on a scale from 0 to 5 (n= 4 per group). (D) Histopathology of mouse brains after intracerebral infection with RABV. Green arrows indicate vascular cuffs; red arrows indicate neuronal necrosis; yellow arrows indicate gliosis. (E) Dynamic changes of serum neutralizing antibody levels in RABV-infected mice (n= 3 per group). (F) Number of differentially expressed genes at different time points based on bulk RNA-seq. [file Image1.tif]

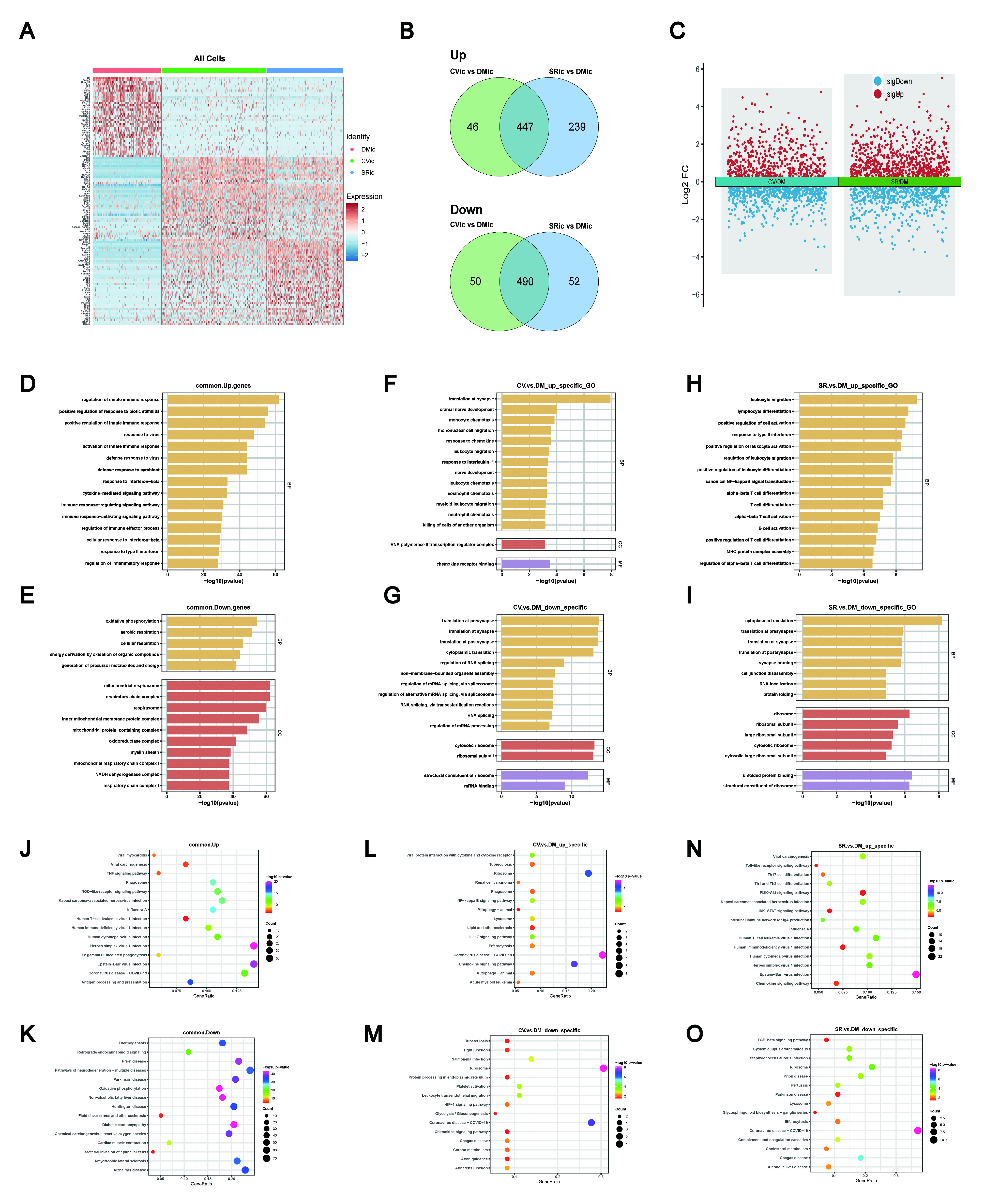

Supplement: Supplementary Figure 2 — Global gene expression changes in mouse brains after RABV infection. (A) Top 10 differentially expressed genes in each group. (B) Overlap of upregulated and downregulated differentially expressed genes among different strains. (C) Volcano plots of upregulated and downregulated differentially expressed genes for different strains. (D–I) GO pathway analysis of upregulated and downregulated differentially expressed genes for different strains. (Yellow: Biological Process; Red: Cellular Component; Purple: Molecular Function.). (J-O) KEGG pathway analysis of upregulated and downregulated differentially expressed genes for different strains. DEGs were defined as those with adjusted P < 0.05 and |log2 fold change| > 0.25. GO and KEGG enrichment analyses were performed using clusterProfiler (version 4.2.2) with hypergeometric test and BH correction; terms with adjusted P < 0.05 were considered statistically significant. [file Image2.tif]

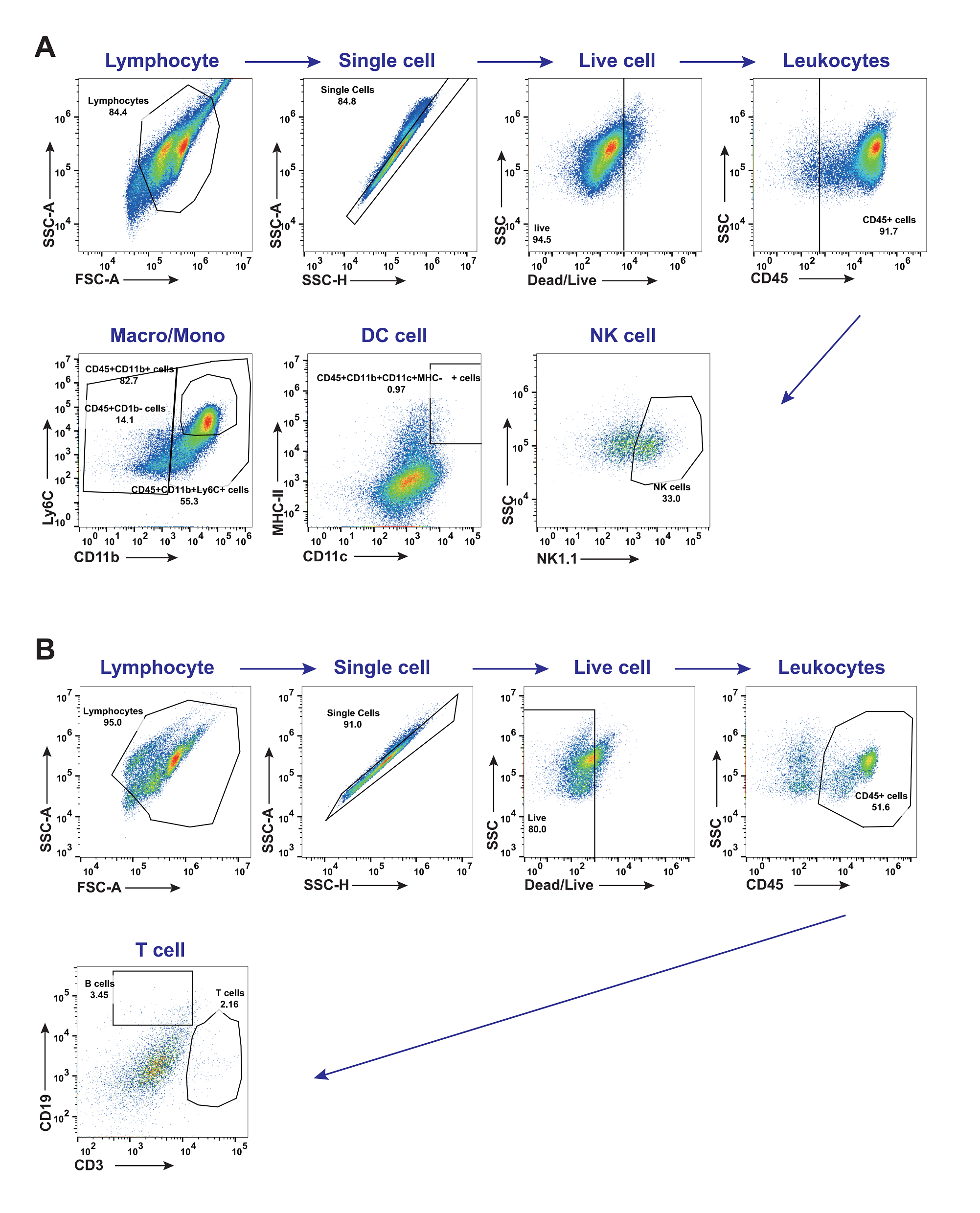

Supplement: Supplementary Figure 3 — Flow cytometric gating strategy for immune cell subsets in mouse brain. (A) Sequential gating workflow for identifying single live CD45+ leukocytes, followed by the separation of CD11b+ myeloid subsets (monocytes/macrophages, DCs) and NK cells based on the expression of CD11b, Ly6C, CD11c and MHC-II. (B) Gating strategy for sorting CD3+ T cells and CD19+ B cells within lymphocyte populations. [file Image3.tif]
